# Task shifting for point-of-care early infant diagnosis: a comparison of the quality of testing between nurses and laboratory personnel in Zimbabwe

**DOI:** 10.1186/s12960-020-0449-2

**Published:** 2020-01-28

**Authors:** Francis M. Simmonds, Jennifer E. Cohn, Haurovi W. Mafaune, Tichaona H. Nyamundaya, Agnes Mahomva, Addmore Chadambuka

**Affiliations:** 1 Elizabeth Glaser Pediatric AIDS Foundation, Block 5-1st Floor, Arundel Office Park, 107 Norfolk Road, Mount Pleasant, Harare, Zimbabwe; 2 Ministry of Health and Child Care, Harare, Zimbabwe; 3 Elizabeth Glaser Pediatric AIDS Foundation, Geneva, Switzerland

**Keywords:** Task shifting, Point-of-care, Tester, Early infant diagnosis, Internal quality controls, Turnaaround time

## Abstract

**Background:**

To decentralize point-of-care early infant diagnosis (POC EID), task shifting to cadres such as nurses is important. However, this should not compromise quality of testing through generating high rates of internal quality control (IQC) failures and long result turnaround times. We used data from a POC EID project in Zimbabwe to compare IQC rates and result return to caregivers for samples run on a POC EID technology (Alere q HIV 1/2 Detect) between nurses and laboratory-trained personnel to assess effects of task shifting on quality of testing.

**Methods:**

This cross-sectional retrospective study used data from all 46 sites (10 hub and 36 spoke sites in Zimbabwe that piloted POC EID for routine clinical use from December 2016 to June 2017). IQC failure rates were downloaded from each POC EID platform and exported to excel to analyze IQC failure rates by type of operator. Turnaround time (TAT) from sample collection to issuing of results to caregiver was extracted from the EID test request form and uploaded into a project specific Excel-based database for analysis.

**Results:**

A total of 1847 tests were conducted by 45 testers (12 laboratory-trained and 33 non-laboratory-trained personnel), including 165 errors. There were no significant differences in IQC failure rates between non-laboratory testers (137 [9.2%] of 14830 tests) and specialized laboratory-trained (28 [7.7%] of 364 tests; *p* = 0.354). Over time, IQC failure rates for both non-laboratory (*χ*^2^ = 18.5, *p* < 0.000) and specialized laboratory-trained testers (*χ*^2^ = 8.7, *p* < 0.003) decreased significantly. There were similar proportions of clients who were issued with results between samples processed by non-laboratory testers (1283 [98.9%] of 1297 tests) and samples processed by specialized laboratory-trained testers (315 [98.7%] of 319 tests; *p* = 0.790). The overall median turnaround time from sample collection to receipt of results by caregiver for samples run by laboratory-specialized testers was not statistically different from samples run by non-laboratory-specialized testers (1 day [IQR 0–3] versus 0 days [IQR 0–2]; *p* = 0.583).

**Conclusions:**

Similar IQC failure rates and TATs between non-laboratory and specialized laboratory-trained operators suggest that non-specialized laboratory-trained personnel can perform POC EID equally well as specialized laboratory personnel.

## Background

Pediatric HIV resulting from mother-to-child transmission (MTCT) continues to be a significant global health problem. Although there have been improvements in prevention of mother-to-child transmission (PMTCT) of HIV, in 2017 approximately 180,000 children were infected with HIV globally [1]. Without rapid diagnosis and early initiation on antiretroviral therapy (ART), 10 to 15% of children infected during pregnancy will die by the age of 6 weeks, 50% by the age of 2 years, and 80% by the age of 5 years [2]. Research has shown that early infant diagnosis and early treatment has the potential to reduce progression of HIV by 75% and early infant mortality by 76% [3]. As such, survival of HIV-infected infants highly depends on early HIV testing, prompt return of test results, and urgent initiation of ART [4]. Point-of-care (POC) early infant diagnosis (EID) of HIV allows for sample analysis at a peripheral health facility thereby improving access to testing and significantly reducing turnaround time (TAT) from sample collection to issuing of results to caregivers [5].

Unlike conventional testing that requires specialized laboratory-trained personnel, POC EID is near-fully automated and may be operated by non-specialized laboratory personnel. Traditionally, operation of all equipment-based diagnostics has been reserved for specialized laboratory personnel. Some earlier studies have concluded that while a qualification with a laboratory training background is more important for one to operate complex devices, for simpler automated devices like those used for point-of-care technology (POCT), performance of operators is independent of their educational level, on condition that a standardized training program is administered prior to conducting any tests [6, 7]. Thus, POC EID allows for task shifting which is a process of delegation whereby appropriate tasks are moved to less specialized health workers [8, 9]. This provides a sustainable and viable solution for EID coverage, especially in resource-constrained settings such as Zimbabwe, as the process ensures more efficient use of the human resources already available [8]. Some studies have actually proved task shifting to increase access to HIV/AIDS services [10] and significantly increase efficiencies in service delivery [11]. Task shifting has the capacity to produce equivalent or superior outcomes for many health interventions including HIV/AIDS [12–14]. However, in some cases, it has been shown that task shifting compromises quality of care, particularly in the absence of close monitoring of some task shifting initiatives [15, 16].

POC EID technologies include internal quality controls (IQCs) that detect user errors. High rates of IQCs may suggest inadequate user capacity. We used data from the POC EID pilot phase in Zimbabwe to compare the IQC failure rates and result return to caregivers for samples run on a POC EID technology (m-PIMA HIV 1/2 Detect) between nurses and specialized laboratory-trained personnel to assess user competence and the feasibility of task shifting.

## Methods

### Study design

We conducted a cross-sectional retrospective study using a secondary data analysis where we compared IQC failure rates and result return to caregivers for samples run on a POC EID technology (m-PIMA HIV 1/2 Detect) between non-laboratory-trained personnel and specialized laboratory-trained personnel to assess user competence.

### Study sites

For the POC EID pilot phase, 10 m-PIMA HIV 1/2 Detect platforms were placed in 2 steps to serve 46 sites in a hub and spoke model, where a hub is a central testing site and a spoke is a peripheral referral site. Tested samples from 10 hub or testing sites and 36 spoke or referral sites were included in this analysis. In consultation with the health facility executive, platforms or machines were either placed in the maternal, neonatal, and child health department (MNCH) or in the laboratory. Testing sites were selected after considering sites that had a historical EID demand of at least 11 tests per month. Spoke sites were added from surrounding facilities that could send samples to the hub site within 24 h from sample collection to further increase access to POC EID. Of the 10 platforms, 6 were placed in MNCH settings where they were operated solely by non-laboratory staff, 2 in the laboratory settings where they were operated solely by specialized laboratory-trained personnel, and 2 in the laboratory settings where they were operated by both cadres.

### Participants

We analyzed data on tests processed by 45 testers (33 non-laboratory personnel and 12 specialized laboratory-trained personnel) between December 2016 and June 2017 from all 46 sites (10 testing sites and 36 spoke sites) in Zimbabwe providing POC EID or near POC EID for routine clinical use.

### Measures

For our first outcome variable “Outcome of test,” data were downloaded from each POC EID m-PIMA HIV 1/2 Detect platform as an Excel file and predictor variables were generated. Using the ID of tester and the training register, we generated a variable “Type of tester.” Each facility had 3 to 5 testers trained. Based on the number of tests done at the end of the pilot phase implementation, we used the date sample processed and the ID of tester to sequentially number the tests for each tester starting from 0 for the first test to generate a proxy variable for experience in POC EID testing “Number of previous tests conducted.” We then classified less than 20 tests as inexperienced users, 20–59 tests as low experience, 60–99 tests as medium experience, and at least 100 tests as high experience. We transformed this variable into a categorical variable showing levels of experience based on the graphical plot for IQC failure rate and number of previous tests conducted. Our final predictor variable was “Type of health facility” generated by identifying the level of facility where the sample was collected from. For our second proxy variable for quality of testing “Turnaround time (TAT) from sample collection to issuing of results to caregiver,” we extracted data from the EID test request form and uploaded into an Excel-based database and merged with the data from the m-PIMA platforms to match samples with the same sample ID number so as to analyze TAT and our predictor variables “Type of tester” and “Type of health facility.”

### Laboratory methods

As part of POC EID routine program implementation, samples were collected directly into an m-PIMA cartridge at testing sites and into a microvette EDTA tube at spoke sites, triple packaged, and transported to a testing site within 72 h. All tests were processed using an Abbott m-PIMA HIV 1/2 Detect platform which is WHO prequalified, CE marked, and got registered and approved for routine clinical use in country. All platform operators were trained and certified by Abbott and received monitoring and supportive supervision 2 weeks, 6 weeks, and 12 weeks post platform installation as required in the POC EID-approved implementation protocol MRCZ/A/2118. In addition, all routine testers passed in an external quality assurance (EQA) program implemented by Zimbabwe National Quality Assurance Program (ZINQAP). ZINQAP facilitated shipment of EQA panels and return of test results and reports from the Public Health Agency of Canada’s Quality Assessment and Standardization of Indicators for Early Infant Diagnosis (QASI-EID) Program.

### Analysis

Categorical variables were summarized by use of frequencies and percentages. Medians and IQRs were used to summarize turnaround times which were continuous variables. For comparative analysis on IQC failures and issuing of results between non-laboratory testers and laboratory specialized testers, we used the *χ*^2^ test. Median turnaround times were compared between non-laboratory testers and laboratory specialized testers by use of the Wilcoxon rank-sum test. Binary logistic regression was used to predict the odds of an IQC failure based on the values of the independent variable. We conducted another binary logistic regression model after controlling for clustering by only considering tests that were at the lower bound of each level of experience. Statistical analysis was done using STATA 12.1.

## Results

Of the 1847 tests included in the analysis, 80.3% were processed by non-laboratory personnel (Table 1). More than half (54.1%) of all tests were for infants identified at testing sites, as opposed to spoke sites. 36.3% of all tests were conducted by inexperienced testers, and 40.5% were conducted by low experienced testers. Medium experienced and highly experienced testers processed 11.7% and 11.5% tests respectively.

**Table 1:** Characteristics of tests run

Test characteristic	Number (%) *N* = 1847
Outcome of test	
Successful	1 682 (91.1)
IQC failure	165 (8.9)
Type of tester	
Laboratory specialized	364 (19.7)
Nurse	1 483 (80.3)
Type of health facility	
Testing site	1 000 (54.1)
Spoke site	847 (45.9)
Number of previous tests run	
< 20 (no experience)	670 (36.3)
20–59 (low experience)	748 (40.5)
60–99 (medium experience)	216 (11.7)
≥ 100 (highly experienced)	213 (11.5)
Site-tester categories	
Laboratory specialized only	253 (13.7)
Nurse only	1 251 (67.7)
Both nurse and laboratory specialized	343 (18.6)

In the comparative analysis (Table 2), there was no significant differences in IQC failure rates between non-laboratory testers (137 [9.2%] of 14830 tests) and specialized laboratory-trained (28 [7.7%] of 364 tests; *p* = 0.354). However, low experienced testers had significantly lower IQC failure rates (47 [6.3%] of 748 tests) compared to inexperienced users (89 [13.3%] of 670 tests; *p* < 0.001). Highly experienced testers also had significantly lower IQC failure rates than those with inexperienced users (7 [3.3%] versus 89 [13.3%] of 670 tests, respectively; *p* = 0.000). However, there was no significant variation between medium experienced testers (22 [10.2%] of 216 tests) and experienced testers (47 [6.3%] of 748 tests; *p* = 0.232).

**Table 2:** Effect of test characteristic on quality of test (either successful or an IQC failure)

Test characteristic	Successful test (%) *N* = 1682 (91.1)	IQC failure (%) *N* = 165 (8.9)	*p* value
Type of tester			
Laboratory specialized	336 (92.3)	28 (7.7)	
Nurse	1 346 (90.8)	137 (9.2)	0.354
Type of health facility			
Testing site	899 (89.9)	101 (10.1)	
Spoke site	783 (92.4)	64 (7.6)	0.056
Number of previous tests run			
< 20 (no experience)	581 (86.7)	89 (13.3)	Ref
20–59 (low experience)	701 (93.7)	47 (6.3)	0.000
60–99 (medium experience)	194 (89.8)	22 (10.2)	0.232
≥ 100 (highly experienced)	206 (96.7)	7 (3.3)	0.000
Site-tester categories			
Laboratory specialized only	240 (94.9)	13 (5.1)	Ref
Nurse only	1 140 (91.1)	111 (8.9)	0.065
Both nurse and laboratory specialized	302 (88.0)	41 (12.8)	0.007

**Fig. 1:**
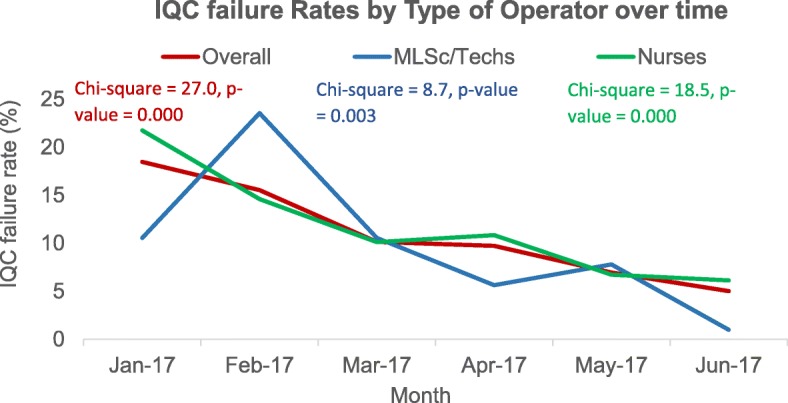
IQC failure rate by type of operator over time

Over time, IQC failure rates for both non-laboratory (*χ*^2^ = 18.5, *p* = 0.000) and specialized laboratory-trained testers (*χ*^2^ = 8.7, *p* < 0.003) decreased significantly (Fig. 1).

In terms of issuing results, there were similar proportions of clients who were issued with results between samples processed by nurse testers (1283 [98.9%] of 1297 tests) and samples processed by specialized laboratory-trained testers (315 [98.7%] of 319 tests; *p* = 0.790) (Table 3). The overall median turnaround time from sample collection to receipt of results by caregiver for samples run by laboratory-specialized testers was not statistically different from samples run by nurse testers (1 day [IQR 0–3] versus 0 days [IQR 0–2]; *p* = 0.583). Turnaround times from sample collection to processing and from sample processing to issuing results to caregiver were not significantly different between tests run by laboratory and nurse testers (0 days [IQR 0–1] versus 0 days [IQR 0–0]; *p* = 0.541, 0 days [IQR 0–2] versus 0 days [IQR 0–1]; *p* = 0.565) respectively.

**Table 3:** Issuing of results by type of operator

Variable	Laboratory specialized (*N* = 319)	Nurse (*N* = 1297)	*p* value
Proportion of results issued to clients	315 (98.7%)	1 283 (98.9%)	0.790
Median time from sample collection to receipt of results by caregiver	1.0 (0.0–3.0)	0.0 (0.0–2.0)	0.583
Median time from sample collection to processing	0.0 (0.0–1.0)	0.0 (0.0–0.0)	0.541
Median time from processing to issuing of results to caregiver	0.0 (0.0–2.0)	0.0 (0.0–1.0)	0.565

Further analysis revealed that in terms of issuing results to caregivers within 14 days (the POC EID project implementation standard), nurse testers (1235 [96.3%] of 1283 tests) had a significantly higher proportion of results issued within 14 days from sample collection compared to specialized laboratory-trained testers (282 [89.5%] of 315 tests; *p* = 0.000) (Table 4).

**Table 4:** Effect of test characteristic on quality of test (either TAT ≤ 14* or TAT > 14 days)

Test characteristic	Number with TAT ≤ 14 days (%) *N* = 1517 (94.9)	Number with TAT > 14 days (%) *N* = 81 (5.1)	*p* value
Type of tester			
Laboratory specialized	282 (89.5)	33 (10.5)	
Nurse	1 235 (96.3)	48 (3.7)	0.000

*14 days is the POC EID program standard

In the logistic regression, experience of tester was independently inversely associated with the probability of an IQC failure (*p* = 0.000) (Table 5). An improvement in the level of experience brings about a decrease in in the odds of producing an IQC failure by roughly a factor of 2.5. Type of tester (*p* = 0.160) and type of health facility (*p* = 0.383) were not significantly associated with IQC failure.

**Table 5:** Logistic regression for an IQC failure

	Log odd ratio Estimates	Odds ratio	95% confidence interval Odds		Pr(>IZI)
			2.50%	97.50%	
Intercept	− 2.285	0.102	0.065	0.158	0.000
Type of tester	0.323	1.381	0.880	2.167	0.160
Type of health facility	− 0.153	0.858	0.608	1.211	0.383
Experience of tester	− 0.434	0.648	0.530	0.792	0.000

After controlling for clustering, type of tester (*p* = 0.638) and type of health facility (*p* = 0.564) remained not statistically significant (Table 6).

**Table 6:** Logistic regression for an IQC failure after controlling for clustering

	Log odd ratio Estimates	Odds ratio	95% confidence interval Odds		Pr(>IZI)
			2.50%	97.50%	
Intercept	− 1.885	0.152	0.030	0.766	0.022
Type of tester	0.406	1.502	0.277	8.149	0.638
Type of health facility	− 0.486	0.615	0.118	3.208	0.564

## Discussion

Our study revealed no differences in IQC failure rates between nurse testers and specialized laboratory-trained testers. This finding corroborates well with Nanji et al [6] who reported that for equipment-based near-patient testing, competency is independent of user laboratory qualifications. This is because POCT platforms used for EID are simpler automated devices and have few operational steps. However, a site with both nurse and laboratory specialized trained testers had significantly high IQC failure rates compared to sites with laboratory specialized trained testers only. This could suggest the need to have the platform operated by one group of cadres to instill a sense of responsibility. In the case where nurse-trained cadres do the testing, laboratory-trained personnel can then be providing backstop support as they are the custodians of all laboratory equipment and not necessarily perform the testing. As IQC failure rates decreased over time possibly due to frequent support and monitoring, more emphasis should be put on the need for a standardized training program to be administered prior to conducting any tests and frequent support and monitoring [6].

Our results also show that nurse-operated POC EID testing will ensure decentralization and timely return of test results without compromising the quality of testing. However, in line with Briggs et al. who asserted that near-patient testing outside laboratory settings can result in dramatic improvements in turnaround time [17], we were expecting a significantly shorter TAT for samples run by nurse-trained testers compared to those run by laboratory-trained testers. Although the TAT for results return was longer for laboratory-specialized testers, overall the percentage of results returned was not different and it is encouraging to see that result communication between laboratory-trained testers and clinicians was rapid and reliable and did not compromise return of results in this POCT model.

We also found that for both nurse testers and specialized laboratory-trained personnel, IQC failure rates decreased significantly over time. Bivariate analysis also showed a significant inverse relationship between number of previous tests conducted and IQC failure rate. Thus, our data demonstrate that with experience comes competency.

Although we used IQC failure rates as a proxy indicator for user competency in this evaluation, IQC rates themselves are important. High rates of IQC failure cause some economic and programmatic inefficiencies. The cartridge for POC EID testing is single use only and IQC failure implies wastage of resources, particularly in resource-constrained settings such as Zimbabwe that heavily depend on donor funds which have been dwindling over the years. POC EID uses whole blood, in some cases from a heal prick directly collected into the cartridge. This means that an IQC failure often results in multiple pricking of an infant, causing unnecessary pain. An m-PIMA HIV 1/2 Detect platform processes one test at a time such that an IQC failure may increase the turnaround time for result communication. This in turn negates the purpose of implementing POC EID which is to ensure timely access to results for HIV-exposed infants. The low rates of IQC failures across laboratory-trained and nursing staff was reassuring that task shifting will not reduce value for money of POC EID.

## Conclusions

Similar IQC failure rates and TATs between non-laboratory and nurse-trained operators suggest that non-specialized laboratory-trained personnel can perform POC EID equally well as specialized laboratory personnel. Nurse-operated POC EID testing will ensure decentralization and timely return of test results without compromising the quality of testing.

### Limitations

Due to the use of secondary data, we could not make use of some tester demographic characteristics like level of education and number of years of work experience as collection of such data was not covered by the IRB approval. However, we did not expect the number of years of work experience to have an effect on quality of testing as the POC EID was a new innovation in the country and no one had been exposed to it.

## Data Availability

The data used and/or analyzed during the study are available from the corresponding author on reasonable request.

## Abbreviations

EGPAF: Elizabeth Glaser Pediatric AIDS Foundation
IQC: Internal quality control
MNCH: Maternal, neonatal, and child health
MOHCC: Ministry of Health and Child Care
MRCZ: Medical Research Council of Zimbabwe
PMTCT: Prevention of mother-to-child transmission
POC EID: Point-of-care early infant diagnosis
TAT: Turnaround time
ZINQAP: Zimbabwe National Quality Assurance Program
